# Classical pathway activity C3c, C4 and C1-inhibitor protein reference intervals determination in EDTA plasma

**DOI:** 10.11613/BM.2019.030707

**Published:** 2019-10-15

**Authors:** Benjamin Lopez, Nicolas Bertier, Emmanuel Ledoult, Romane Joudinaud, Mehdi Maanaoui, Victoria Majerus, Emmanuelle Moitrot, Anne-Sophie Deleplancque, Stéphanie Rogeau, David Launay, Guillaume Lefèvre, Myriam Labalette, Sylvain Dubucquoi

**Affiliations:** 1 Department of Immunology, Lille University Hospital, Lille, France; 2 Lille Inflammation Research International Center, University of Lille, Lille, France; 3 Department of Internal Medicine and Clinical Immunology, Lille University Hospital, Lille, France; 4 Department of Nephrology, Lille University Hospital, Lille, France; 5 National Reference Center for Angioedema (CREAK), Grenoble, France

**Keywords:** reference values, complement C3c, complement C4, classical complement pathway, complement C1 inhibitor protein

## Abstract

**Introduction:**

Reference intervals (RIs) for complement assays in EDTA plasma samples have not previously been published. The objectives of the present study were to validate and/or determine RIs for classical pathway (CP50) activity and C3c, C4 and C1 inhibitor protein (C1INH) assays and to assess the need for age-specific RIs in EDTA plasma.

**Materials and methods:**

We retrospectively evaluated a cohort of 387 patients attending our university hospital and known to be free of complement-modifying diseases. The need for age partitioning was assessed and RIs were calculated according to the CLSI protocol.

**Results:**

No need for age partitioning was evidenced for CP50 activity, C3c and C4 concentrations and RIs (90% CI) were calculated from the pooled data: 35.4 (33.1-37.2) to 76.3 (73.7-83.6) U/mL for CP50 activity, 0.80 (0.75-0.87) to 1.64 (1.59-1.72) g/L for C3c, and 0.12 (0.10-0.14) to 0.38 (0.36-0.40) g/L for C4. Our results highlight a positive association between age and C1INH concentrations. We derived 3 age partitions (6 months to 30 years, 30-50 and > 50 years) and the related RIs: 0.20 (0.18-0.21) to 0.38 (0.36-0.40) g/L, 0.22 (0.20-0.24) to 0.39 (0.36-0.41) g/L and 0.25 (0.22-0.27) to 0.41 (0.40-0.43) g/L, respectively).

**Conclusions:**

The newly determined RIs for CP50 activity were higher than those provided by the manufacturer for EDTA plasma samples, whereas those for C3c and C4 RIs were similar to the values provided for serum samples. The C1INH concentration and activity were found to be associated with age and age-specific RIs are mandatory for this analyte.

## Introduction

In clinical practice, the assessment of complement activity and complement component protein concentrations is an essential part of the diagnostic process for a variety of disease, ranging from potential life-threatening primary immunodeficiencies to inflammatory diseases and autoimmune disorders caused by over-activation of the complement system (*1*). Consequently, assays of C3c, C4 and C1-esterase inhibitor (C1INH) proteins and classical pathway activity (CP50) are widely used in clinical laboratories as first-line complement screening tools, prior to referral (if required) to a specialist unit (*1*, *2*).

At present, most clinical laboratories have moved from older, haemolysis-based (CH50) classical pathway activity assays to automated, large-batch and high-throughput assays for their routine analyses. For example, automated single-point liposomal CP50 assays (with no need serial sample dilutions) are now quite common (*1*, *3*). Furthermore, many nephelometric or turbidimetric assays are now commercially available for the easy, routine determination of specific complement proteins such as C3c, C4 and C1INH (*1*). However, the predominant use of polyclonal antibodies in these assays makes them potentially sensitive to breakdown products generated by *in vitro* complement activation (*3*).

The preanalytical phase has always been a challenging part of the complement assay process. Indeed, inappropriate sample collection or handling may lead to *in vitro* complement activation and thus the generation of biased or inconclusive results (*2*, *4*). Mollnes *et al*.’s pioneering study showed that the use of EDTA-containing sample collection tubes and refrigerated (4 °C) sample storage are both effective ways of limiting inappropriate complement activation (*5*). These requirements appear to be even more critical for complement activation product assays (*1*). More recently, Yang *et al*. concluded that these preanalytical procedures stabilized the sample for up to 24 hours (*6*). Surprisingly, these well-accepted requirements have not been widely applied and many laboratories still perform complement assay on serum samples. For example, there are currently no plasma-based external quality assessment programs for CP50 activity and C3c, C4 and C1INH protein assays. Nevertheless, many assays have been validated for the quantification of complement activity and component concentrations in plasma, including the Optilite^®^ assays (The Binding Site Group Ltd, Birmingham, UK) – even though the manufacturer does not supply plasma reference intervals (RIs) for each test.

Hence, the objectives of the present study were to: (i) validate the manufacturer’s recommended RIs for the liposomal CP50 assay, (ii) determine previously lacking RIs for the quantitative turbidimetric C3c, C4 and C1INH assays, in EDTA plasma samples, using the Optilite^®^ analyser and (iii) to assess the need for age-specific RIs for these various parameters.

## Materials and methods

### Subjects

We performed a retrospective study of consecutive inpatients and outpatients (regardless of the admitting hospital department) having been tested for complement parameters in the laboratory at Lille University Hospital’s Department of Immunology (Lille, France) between October 2016 and December 2017. The lower age boundary was 6 months, and there was no upper age boundary. The clinical and laboratory data studied here were extracted from our hospital’s central database, anonymized and then retrospectively reviewed by two investigating physicians who were blinded to the complement assay results. In the event of disagreement on whether or not to exclude a patient, a third investigator reviewed the data.

The main exclusion criterion was the presence of an acute condition (within the previous 3 months) or chronic condition that could have influenced complement concentrations. When available, we used either: i) haematological or biochemical criteria compatible with complement activation or defective synthesis and/or ii) clinical evidence of complement-modifying disorders (*1*, *4*, *7*). Briefly, the criteria covered were: i) diabetes mellitus (glycated haemoglobin (HbA_1c_ > 48 mmol/mol), white blood cell count (WBC) < 4 x 10^9^/L or > 10 x 10^9^/L, an elevated C-reactive protein (CRP > 3.0 mg/L), a markedly elevated concentration of aspartate/alanine aminotransferase or gamma-glutamyltransferase (AST, ALT, GGT > 100 U/L), and the presence of cryoglobulinaemia and ii) evidence of complement dysregulation pathologies or concomitant infectious, inflammatory, autoimmune or neurodegenerative diseases (the various disorders and their corresponding laboratory criteria are listed in Supplementary table 1). The laboratory measurements were either performed on the same day as the complement analyte assay (WBC and CRP concentration) or within the 3-month periods before and after the assay (liver enzymes and HbA_1c_). Some measurements (*e.g*. cryoglobulinaemia) were carried out in a subset of the population only. A lack of detailed laboratory data or clinical records was a study exclusion criterion. The included population was split into a paediatric partition (< 18 years) and an adult partition (≥ 18 years).

Patients included during a routine consultation at Lille University Hospital were informed that their laboratory and clinical data might be subsequently used for research purposes and were given an opportunity to refuse this use. The collection and storage of biological material and the corresponding medical datasets were registered with and authorized by the French Ministry of Research (*No.* DC-2008-642). In line with the regulations set out by the French National Data Protection Commission and international guidelines, written, informed consent was neither required nor requested for this non-interventional study (*8*).

### Methods

All the complement assays were performed as part of the routine workflow at Lille University Hospital’s Department of Immunology. It should be noted that the complement assays’ preanalytical phase was governed by strict requirements: only EDTA plasma samples collected with 6.0 mL Vacutainer tubes (10.8 mg K_2_EDTA, BD Vacutainer, Becton Dickinson, Franklin Lakes, USA) were used. The samples were delivered to the laboratory using a pneumatic transport system within 3 hours of collection at ambient temperature. The whole blood sample was either stored at 4 °C prior to centrifugation, divided into three aliquots and analysed within 24 hours of collection (the complement assays were run monday to friday), or frozen at - 80 °C if the assay was delayed (maximum: 72 hours; one freeze-thaw cycle). This activity has been accredited according to the ISO 15189 standard.

We used CE-marked commercially available Optilite^®^ assays on the fully automated Optilite^®^ turbidimetric analyzer (The Binding Site Group Ltd, Birmingham, UK), according to the manufacturer’s instructions. As part of our department’s routine workflow, each request for complement assays includes measurements of CP50 activity and C3c and C4 protein concentrations - enabling correct interpretation of the results. The C1INH protein assay is performed when specifically requested or when the patient’s only abnormal result is a low C4 concentration. The reagent used routinely to assess CP50 activity is the Optilite CH50 Kit^®^ (product reference: NK095.OPT, batches 361755, 401282, 420391, 401283 and 406914) liposome-based immunoassay, which utilizes dinitrophenyl (DNP)-coated liposomes that contain the enzyme glucose-6-phosphate dehydrogenase (G6PD). Adjunction of the plasma sample and a substrate containing anti-DNP antibodies and G6P activates the liposomes lyse; the resulting enzymatic colorimetric reaction is proportional to total classical complement activity. As there is no reference material for CP50 liposomal assays, it uses a pool of human sera calibrated against an internal reference material. The Optilite C3c Kit^®^ (reference: NK023.OPT, batches 356792, 368552 and 401388), the Optilite C4 Kit^®^ (reference: NK025.OPT, batches 356795, 368554 and 401390) and the Optilite C1 Inactivator Kit^®^ (reference: NK019.OPT, batches 360370, 401387 and 407508) turbidimetric assays were used to measure C3c, C4 and C1INH protein concentrations. Those assays utilize polyclonal antibodies against the targeted analyte to form antigen-antibody complexes, which are measured by turbidimetry. The C3c and C4 protein assays are calibrated against the international reference material ERM-DA470k. The C1INH protein assay uses a pool of human sera calibrated against an internal reference material (*9*). Routinely, two internal quality controls (qualified in-house pooled plasmas, used over one year) are used in parallel with each sample run. The intra-assay coefficients of variation (CVs) obtained from 10 repeated measurements of low and high analyte concentrations were respectively 1.0 and 1.6% for CP50, 3.2 and 1.9% for C3c, 1.7 and 1.1% for C4, and 1.3 and 1.4% for C1INH assays. The inter-assay CVs calculated for routine in-house mean data from internal quality controls (low and high analyte concentrations) over six months were respectively 6.4 and 2.8% for CP50, 8.5 and 9.5% for C3c, 7.6 and 6.2% for C4, and 3.5 and 3.9% for C1INH. These CVs are in line with the manufacturer’s recommendations, the currently available imprecision specifications and the literature data - although the Optilite^®^ assay’s analytical performances have only been published for C3c, C4 and C1INH protein concentrations (*9*, *10*).

The C1INH activity was assessed using a chromogenic assay (Technoclone GmbH, Vienna, Austria) whose diagnostic performances have already been described (*9*). The results are expressed as the percentage of C1INH activity *vs* a reference sample; a normal result corresponds to between 70% and 130%. The intra-assay CVs (obtained from 10 repeated measurements of low and high analyte concentrations) were respectively 4.6 and 5.7%. The inter-assay CVs calculated for routine in-house mean data from internal quality controls (low and high analyte concentrations) over six month were respectively 12.1 and 8.2%.

### Statistical analysis

Statistical analyses were performed using MedCalc^®^ for Windows software (version 17.4, MedCalc Software, Ostend, Belgium). The Shapiro-Wilk test and dispersion indices were used to determine whether the data were normally distributed. P-values < 0.05 were considered statistically significant.

Patient data were selected using a direct sampling/*a posteriori* approach and RIs were determined according to the Clinical and Laboratory Standards Institute (CLSI) EP28-A3c guidelines (*11*, *12*). The calculation method has been described in detail elsewhere (*12*, *13*). When the manufacturer had already published RIs for EDTA plasma samples, we applied the transference validity method described by the CLSI: 20 reference individuals were randomly selected (using the RAND function in Excel^®^ software) from our population and their analyte concentrations were compared with the manufacturer’s RIs (*12*). If more than 2 of the 20 values fell outside the RI, 20 more patients were selected and analysed. Ultimately, the manufacturer’s RI was considered non-transferable if 5 or more patient values fell outside it. With regard to RI determination, outliers were removed using Grubb’s test for subgroups with normally distributed data or after a Box-Cox power transformation and then Tukey outlier detection for subgroups with a skewed data distribution (*12*, *14*). Futhermore, the lower and upper reference limits (the 2.5^th^ and 97.5^th^ percentiles) and the corresponding 90% confidence intervals (CIs) were then calculated using a non-parametric method when the number of observations exceeded 120 or Horn and Pesce’s alternative, robust method when only a small number of observations (N < 120) were available (*12*, *14*). Finally, Harris and Boyd’s test was applied to determine whether the adult and paediatric age partitions were different enough to warrant separation (*12*, *13*). Harris and Boyd’s test compares a calculated z statistic with a critical value z*; if the calculated value exceeds z, then partitioning is recommended (see reference 12 for details of the z statistic calculations). The additional standard deviation criterion (stating that the largest standard deviation should be 1.5 times greater than the smallest standard deviation) was also applied. If none of these criteria was significant, the partitions’ data were pooled, and the RIs were calculated for the newly obtained groups by applying the methodology described above. If at least one of the two criteria were violated, partitioning was considered as mandatory and a weighted polynomial regression was assigned to the mean and standard deviations in order to model the variations in the concentration (using the age-related RI procedure in MedCalc Software) (*15*). Lastly, the appropriateness of the final partitioning was decided by visual inspection of the plots. The precision of the estimated RIs and corresponding CIs was evaluated as the ratio of the width of each lower and upper reference limit’s 90% CI to the RI’s width.

## Results

**Figure 1:**
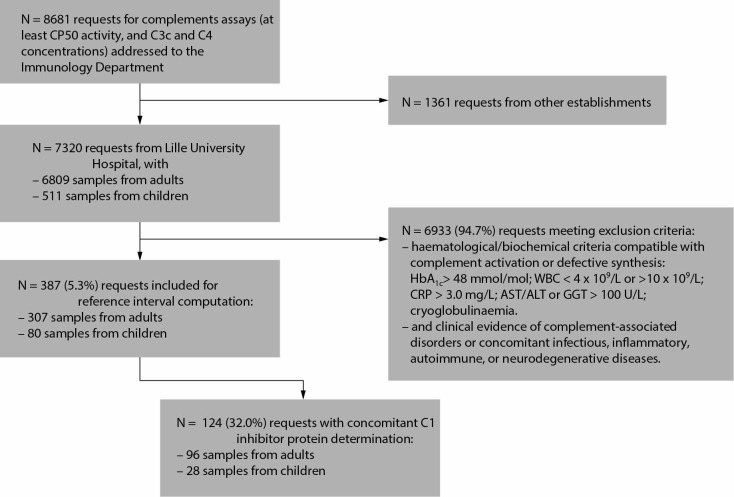
Study flowchart. CP50 - classical pathway activity. HbA_1c_ - glycated haemoglobin. WBC - white blood cell. CRP - C-reactive protein. AST/ALT - aspartate/alanine aminotransferase. GGT - gamma-glutamyltransferase.

During the 15-month inclusion period, 8681 requests for complement assays (with concomitant CP50, C3c and C4 measurements) were sent to the Immunology Department. Of these, 7320 samples (6809 samples from adults and 511 samples from children) came from Lille University Hospital itself - ensuring the availability of corresponding medical records. After application of the exclusion criteria (Supplementary table 1) and the removal of duplicates, 387 samples with data for CP50 activity and C3c and C4 protein concentrations were included in the final analysis. These came from 307 adults and 80 children. A subset of this population also had assay data for C1INH (96 adults and 28 children). The study flowchart is shown in Figure 1 and demographic data on the study populations are summarized in Table 1.

**Table 1:** Demographic data of the included populations

	**Determination of RIs for CP50, C3c and C4** **(N = 387)**		**Determination of RIs for C1 inhibitor** **(N = 126)**	
Subgroups	**Adult patients** **N = 307**	**Paediatric patients** **N = 80**	**Adult patients** **N = 96**	**Paediatric patients** **N = 28**
Females, N (proportion)	179 (0.58)	47 (0.59)	65 (0.68)	19 (0.68)
Age, years	48 (18-88)	14 (0.5-18)	46 (18-93)	13 (0.6-18)
Age is presented as median (range). RI - reference interval. CP50 - classical pathway activity.				

The manufacturer provides a RI for EDTA plasma samples in the CP50 assay. We found that this RI was not transferable since the values were above the upper boundary for 3 and 4 individuals in the first and second randomly selected subsets of 20 patients, respectively. For both age partitions, the CP50 data were normally distributed but the C3c and C4 data displayed lognormal distributions; logarithmic transformation gave normal distributions for both datasets. The tests for outlier screening and RI computations were applied accordingly. The calculated RIs (90% CI) for the adult and paediatric partitions are given in Table 2, together with the number of outliers removed from the included population (N = 387) for each calculation (one value (0.3%) for the CP50 activity and C3c concentration, and three (0.8%) for the C4 concentration). In Harris and Boyd’s test, a comparison of the age-partitioned RIs yielded z statistics that were below the calculated critical value (3.73) for all three parameters: 1.95 for CP50, 0.03 for C3c, and 1.37 for C4. Furthermore, none of the standard deviation ratios exceeded 1.5 (1.16, 1.07, and 0.94 for CP50, C3c and C4, respectively). Hence, the age groups were pooled into a single population for all three parameters. The new partitions and their RIs (90% CI) are shown in Table 2. The precision ratio of the estimated RIs and the corresponding CIs was always below 20%.

**Table 2:** Reference intervals for the classical pathway activity and for C3c and C4 protein concentrations

**Analyte**	**Patients** **(N)**	**Median**	**Lower reference limit (90% CI)**	**Upper reference limit (90% CI)**	**Outliers removed** **(N)**
***Adult partition***					
CP50, U/mL	306	55.0	35.4 (32.4-37.2)	76.4 (73.9-84.9)	1
C3c, mg/L	306	1.16	0.81 (0.72-0.87)	1.64 (1.59-1.80)	1
C4, mg/L	304	0.24	0.13 (0.11-0.14)	0.37 (0.36-0.41)	3
C1-inhibitor, mg/L	96	0.30	0.21 (0.19-0.22)	0.41 (0.39-0.42)	0
***Paediatric partition***					
CP50, U/mL	80	52.7	34.1 (30.7-37.4)	71.4 (68.1-74.6)	0
C3c, mg/L	80	1.16	0.83 (0.79-0.88)	1.61 (1.52-1.70)	0
C4, mg/L	80	0.20	0.11 (0.09-0.12)	0.37 (0.34-0.40)	0
C1-inhibitor, mg/L	28	0.29	0.21 (0.19-0.24)	0.37 (0.34-0.39)	0
***Aggregate partition***					
CP50, U/mL	386	54.4	35.4 (33.1-37.2)	76.3 (73.7-83.6)	0
C3c, mg/L	386	1.16	0.80 (0.75-0.87)	1.64 (1.59-1.72)	0
C4, mg/L	384	0.24	0.12 (0.10-0.14)	0.38 (0.36-0.40)	0
Given that the application of Boyd and Harry’s test prevented us from aggregating the partitions for C1-inhibitor, age-related RIs were calculated (see Table 3). 90% CI – 90% confidence intervals.					

**Figure 2:**
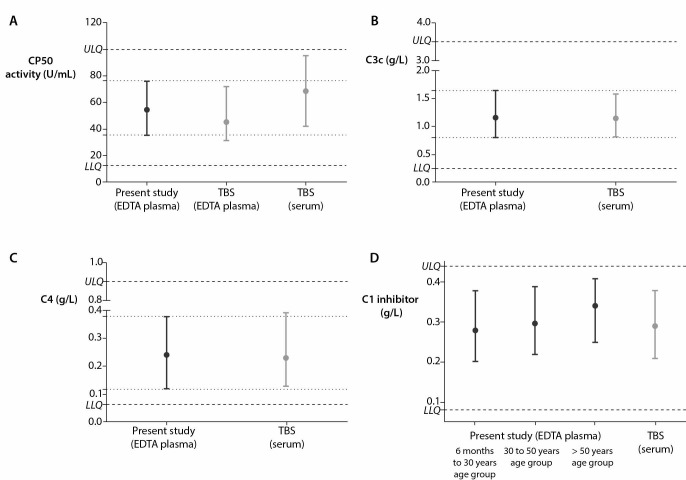
Median and reference intervals (2.5^th^ and 97.5^th^ percentiles) obtained in the present study (dark dots and bars), and those provided by the manufacturer for EDTA plasma samples (when available) and/or serum samples (grey dots and bars). (A) CP50 activity: 35.4 to 76.3 U/mL (present study), 31.7 to 71.4 U/mL (The Binding Site (TBS) for EDTA plasma samples) and 41.7 to 91.1 U/mL (TBS for serum samples). (B) C3c concentrations: 0.80 to 1.64 g/L (present study) and 0.81 to 1.57 g/L (TBS for serum samples). (C) C4 concentrations: 0.12 to 0.38 g/L (present study) and 0.13 to 0.39 g/L (TBS for serum samples). (D) C1 inhibitor protein concentrations: 0.20 to 0.38 g/L (from 6 months to 30 years), 0.22 to 0.39 g/L (30 to 50 years), 0.25 to 0.41 g/L (> 50 years) and 0.21 to 0.38 g/L (TBS for serum samples). CP50: classical pathway activity, LLQ: lower limit of quantification, ULQ: upper limit of quantification. Dashed lines correspond to the LLQ and ULQ. The dotted lines correspond to the RIs determined in the present study.

For CP50 activity, the newly determined RI (90% CI) was 35.4 (33.1-37.2) to 76.3 (73.7-83.6) U/mL, *vs*. 31.7-71.4 U/mL quoted by the manufacturer for EDTA plasma samples (Figure 2A). For the C3c concentration, the newly determined RI was 0.80 (0.75-0.87) to 1.64 (1.59-1.72) g/L, *vs.* 0.81-1.57 g/L quoted by the manufacturer for serum samples (Figure 2B). For the C4 concentration, the newly determined RI was 0.12 (0.10-0.14) to 0.38 (0.36-0.40) g/L, *vs.* 0.13-0.39 g/L quoted by the manufacturer for serum samples (Figure 2C).

The data on C1INH concentrations were normally distributed in both age partitions. No outliers were found in the adult or paediatric partitions. In contrary to the above-mentioned results for CP50 activity and C3c and C4 protein concentrations, the application of Harris and Boyd’s test suggested that the age groups should not be pooled: even though the z statistic (0.41) was below the critical value (2.15), the standard deviation ratio was 1.83; hence, age-specific RIs were determined. The best fit weighted polynomial regression was achieved with the addition of a quadratic term to the equation using C1INH protein concentrations and age as the dependent and independent variables, respectively (*15*). The resulting curve had two distinct parts; it was flat until the age of 30, and then increased with age thereafter. This procedure generated RIs (90% CI) for each decade of life. Ultimately (and as already reported by others), we used a graphical method to decide on the partitions and to derive more practical RIs with narrower CIs (*16*, *17*). Three age groups were built: i) 6 months to 30 years, ii) 30-50 years and iii) > 50 years and RIs were calculated using a robust method. Again, the application of Harris and Boyd’s test suggested that these three age partitions groups should not be pooled and the precision ratios were between 15 and 33%. The median C1INH concentrations and the RIs increased with age. For example, the lower reference limit (90% CI) increased from 0.20 (0.18-0.21) to 0.22 (0.20-0.24) and 0.25 (0.22-0.27) g/L in the three groups. In comparison, the manufacturer’s RI (determined on serum samples from adults) was 0.21 to 0.38 g/L. Detailed results are provided in Table 3 and Figure 2D.

**Table 3:** Reference intervals for C1 inhibitor protein concentration

**Age partition**	**Patients** **(N)**	**Median**	**Lower reference limit (90% CI)**	**Upper reference limit (90% CI)**	**Outliers removed** **(N)**
6 months to 30 years	56	0.28	0.20 (0.18-0.21)	0.38 (0.36-0.40)	0
30 to 50 years	35	0.30	0.22 (0.20-0.24)	0.39 (0.36-0.41)	0
> 50 years	33	0.34	0.25 (0.22-0.27)	0.41 (0.40-0.43)	0
The concentrations of C1-inhibitor protein are expressed in mg/L. 90% CI – 90% confidence intervals.					

To further validate our findings, we analysed the functional C1INH activity data that were available for a subset (N = 89) of the C1INH population. None of the patients in our study population had a C1INH activity value below 70%; the lowest measured value was 83%. In the present analysis, all the values above the measurement range (> 150% of the normal control value) were censured to 150%. A simple linear regression analysis revealed a positive association between the C1INH protein concentrations and functional activity: the estimates (95% CI) for the slope and intercept were 0.22 (0.17-0.26) and 51 (37.2-64.8), respectively (P < 0.001), with a determination coefficient of r^2^ = 0.53. All the assumptions for linear regression were met. Furthermore, a weighted polynomial regression similar to the one previously described yielded the same two-segment pattern as for C1INH protein concentrations (data not shown).

## Discussion

Using Optilite^®^ turbidimetric assays, we determined CP50 activity and C3c, C4 and C1INH protein concentrations in EDTA plasma samples from 387 in- and outpatients aged from 6 months to 88 years and who were free of complement-modifying disorders. No age partitions were needed for CP50 activity, C3c and C4 protein concentrations; hence, RIs were calculated from pooled data. In contrast, C1INH protein concentrations were positively associated with age and so required age partitioning.

The use of EDTA plasma samples effectively minimizes preanalytical interference, enhances analyte stability and thus ensures the results’ reliability (*6*). The biochemical rationale behind EDTA’s efficiency relates to the blockade of the complement cascade through calcium removal. It is noteworthy that in 2013, the International Complement Standardization Committee produced an international standard for measuring complement activation products (*18*). The activation of serum samples was stopped effectively by the adjunction of both EDTA and nafamostat-mesylate and the resulting complement activation products were remarkably stable.

Activity of CP50 and C3c and C4 protein concentrations have been studied extensively in term and pre-term new-borns and in children: for all three parameters, it is thought that adult concentrations are reached within 3-6 months of life (*19*, *20*). Hence, we chose not to include patients younger than 6 months. No need for partitioning was observed in the present study. Likewise, in a recent determination of paediatric RIs for serum complement components using Optilite^®^ reagents, Garcia-Prat *et al.* did not evidence any age-related differences in C3c and C4 protein concentrations (*21*). Relative to the manufacturer’s RIs for EDTA plasma, we found higher concentrations for CP50 activity (Figure 2A). It is noteworthy that the manufacturer’s proposed RI (for which a CI was not published) fell outside our newly calculated 90% CI. Our findings are in line with the RIs calculated by Yoon *et al.* for another liposome-based immunoassay (Wako, Osaka, Japan), although the difference between the manufacturer’s RI and the newly determined RI was greater in the latter study than in our study. In Yoon *et al.*’s study, the newly calculated RIs were also higher than the values noted by the manufacturer and yielded a more accurate classification (normal *vs.* pathological) in a small validation cohort (*7*). Although the application of our newly determined RI will doubtless change the classification of patients as having normal or abnormal levels of CP50 activity, a lower proportion of patients will likely be concerned by this change (relative to Yoon *et al.*’s values).

For C3c and C4 proteins, as no data were available for plasma samples, our RIs appear to be very similar to those provided by the manufacturer for serum samples. To the best of our knowledge, the present study is the first to have provided plasma sample RIs for C3c and C4 protein concentrations.

Unexpectedly, we found an age-related rise in the median concentrations and RIs for C1INH in all three age groups. In the present study, the observed parallel increase in C1INH concentrations and activity with age strengthens our results. Data on variations in the C1INH concentration with age are scarce – especially with regard to changes in C1INH concentrations during childhood and thereafter (*19*, *20*). Andrew *et al.*’s study of variations in component concentrations during childhood reported that C1INH concentrations were highest in the first few years of life and then declined until adulthood (*22*). These findings contradict our results and reports that both preterm and term neonates have lower C1INH protein concentrations and activity than in adult patients (*23*). The small number of young infants in the present study might have influenced the age distribution of C1INH concentrations, although the available values were similar to those determined in young adults.

As for C3c and C4, the C1INH RIs determined here for the 30-50-year age partition were close to the published values for serum samples (0.21 to 0.38 g/L in healthy adult blood donors) (*9*).

The most common disease entities associated with C1INH deficiency are hereditary angioedema type I (low protein concentrations and low activity) and type II (normal/elevated protein concentration but low activity) (*24*, *25*). One of the diagnostic criteria for type I angioedema is a C1INH protein concentration below 50% of the normal value (*24*). Furthermore, testing must always be repeated for a reliable diagnosis (*24*, *25*). Hereditary angioedema with C1 inhibitor deficiency is generally diagnosed in children or young adults, with symptom onset before the age of 20 (*24*, *25*).

Our results fit well with the “inflamm-aging” concept that has emerged over the last 20 years or so (*26*). This concept is based on a growing body of evidence for a chronic, low-grade inflammatory state that progresses with age. Increasing concentrations of circulating cytokines and pro-inflammatory markers (*e.g*. interleukin (IL)-6, IL-1, and tumour necrosis factor-α) have been reported, and appear to be linked to many age-related diseases (*26*, *27*). Hence, increasing concentrations and functional activities of anti-inflammatory molecules with age are of particular interest as a physiological means of countering this chronic, low-grade inflammation. As our understanding of C1INH’s anti-inflammatory properties has improved, it has been possible to initiate C1 replacement therapy in severe sepsis (in which C1INH deficiencies have been reported) (*28*). In this context, and given the high variability within patient populations, age-specific RIs might be of use in ongoing clinical trials.

The limitations of this study were that we initially performed the transference validation procedure (as described in the CLSI EP28-A3c document) using the manufacturer’s reported RIs for the liposomal CP50 assay with EDTA plasma samples; our results showed that these RIs were not transferable. No RIs were available for C3c, C4 and C1 inhibitor measurements in EDTA plasma, and a transference study was prevented by the absence of bias estimation data generated using different methods. Hence, we determined new RIs for liposomal CP50 activity and C3c, C4 and C1 inhibitor concentrations in EDTA plasma samples. The current CLSI guidelines do not endorse RI calculation techniques based on indirect sampling, *i.e*. studies of both diseased and non-diseased subjects in which the reference population is identified through sole statistical methods alone – even though this approach was advocated by a recent opinion paper on behalf of the International Federation of Clinical Chemistry (*11*, *12*). Even though we performed a retrospective study, we therefore decided to use an *a posteriori* direct sampling approach, defined as one in which “specimens collected from a population will be included in the analysis based on other factors such as clinical details or other measurement results, which were not used to define the collection.“ (*11*). Given that our study participants were selected from a broad range of hospital departments, the careful analysis of medical records and laboratory data was essential for ruling out a potential recruitment bias. Out of an initial population of 7320 eligible patients with complement component assays, only 387 (5.3%) met all of our inclusion criteria and none of our exclusion criteria. We believe that the relatively small size of this proportion attests to the rigorousness of our inclusion process. There are no clear guidelines on how to manage analytes whose RIs change continuously with age are not available (*12*, *29*). Overall, the 90% CIs of the upper or lower reference limits for CP50 activity and C3c and C4 protein concentrations were not excessively broad. In contrast, and despite a total population of 124 patients, our partitioning decisions led to small numbers of patients in each age group for C1INH. Hence, the 90% CIs were broad for almost all the C1INH RIs, and the RIs suggested here must be considered with a degree of caution. Large numbers of patients are needed to meet the precision criteria set out in the CLSI EP28-13c document (*12*, *30*). When several age partitions are necessary, this large sample size is hard to obtain.

We determined RIs for Optilite^®^ immunoturbidimetric reagents (commercialized by The Binding Site Group Ltd). The newly determined RIs for CP50 activity were higher than those provided by the manufacturer for EDTA plasma samples, whereas the C3c and C4 RIs were similar to the values provided for serum samples. The C1INH protein concentration and activity were found to be associated with age; hence, age-specific RIs are mandatory for this analyte.

## Supplementary material

Supplementary table 1. Summary of complement-modifying disorders (used as exclusion criteria) and the corresponding haematological or biochemical criteria (adapted from Yoon et al. and Prohászka et al. (*1*, *7*)
