# Supplementary material for: Classical pathway activity C3c, C4 and C1-inhibitor protein reference intervals determination in EDTA plasma
Source: Biochem Med (Zagreb). 2019 Oct 15;29(3):030707. doi: 10.11613/BM.2019.030707 (PMC6784422; doi:10.11613/BM.2019.030707)
Supplement: Supplementary file 1 — Supplementary table 1. Summary of complement-modifying disorders (used as exclusion criteria) and the corresponding haematological or biochemical criteria (adapted from Yoon et al. and Prohászka et al. (1, 7) [file bm-29-3-030707-S1.pdf]

Supplementary table 1. Summary of complement-modifying disorders (used as exclusion criteria) and the corresponding haematological or biochemical criteria (adapted from Yoon et al. and Prohászka et al. (1,7)

Category	Disorders
**Haematological or biochemical criteria**	HbA_{1c} > 48 mmol/mol<br>WBC < 4 x10^9/L or > 10 x10^9/L<br>C-reactive protein > 3.0 mg/L<br>AST/ALT > 100 U/L<br>GGT > 100 U/L<br>Presence of cryoglobulinaemia
**Infectious diseases**	Any acute bacterial, viral or fungal infection (sepsis) or chronic bacterial, viral or fungal infection.<br>Any recurrent infections, particularly those due to encapsulated bacteria.
**Inflammatory diseases**	Rheumatoid arthritis.<br>Any kind of vasculitis.<br>Atherosclerotic vasculitis.<br>Nephritis.<br>Inflammatory bowel diseases.<br>Systemic inflammatory reaction syndrome.<br>Ischemia-reperfusion injury.
**Autoimmune diseases**	Systemic lupus erythematosus.<br>Systemic sclerosis.<br>Anti-phospholipid syndrome.<br>Multiple sclerosis.<br>Myasthenia gravis.<br>Psoriasis.
**Diseases of complement dysregulation**	Atypical haemolytic uremic syndrome.<br>Glomerulonephritis.<br>Paroxysmal nocturnal haemoglobinuria.<br>Hereditary or acquired angioedema
**Neuro-degenerative diseases**	Alzheimer’s disease.<br>Parkinson’s disease.<br>Age-related macular degeneration.
**Others**	Immediate post-operative context.<br>Acute or chronic graft-versus-host reaction or transplant rejection.<br>Stroke.<br>Myocardial infarction.<br>Trauma.<br>Burns.<br>Capillary leak syndrome.

	Biomaterial incompatibility (hemofiltration, plasmapheresis, cardiopulmonary bypass, *etc*.)

HbA_{1c} - glycated haemoglobin. WBC - white blood cell count. AST/ALT - aspartate/alanine aminotransferase. GGT - gamma-glutamyltransferase. Please remove this part from here.
